# Pt–Cr Coated 3D-Printed Porous Transport Layers for Proton-Exchange Membrane Water Electrolyzers Prepared by Electron Beam Evaporation

**DOI:** 10.1021/acs.langmuir.5c06168

**Published:** 2026-03-18

**Authors:** Murat Kıstı, Emre Özdoğan, Sami Pekdemir, Mehmet Fatih Kaya

**Affiliations:** † Heat Engineering Division, Energy Systems Engineering Department, Engineering Faculty, 52958 Erciyes University, 38039 Kayseri, Türkiye; ‡ Energy Systems Engineering Department, Graduate School of Natural and Applied Sciences, 52958 Erciyes University, 38039 Kayseri, Türkiye; § Erciyes University H2FC Hydrogen Energy Research Group, 38039 Kayseri, Türkiye; ∥ 52958 Erciyes University, ArGePark Research Building, 38039 Kayseri, Türkiye; ⊥ Department of Aeronautical Engineering, Faculty of Aeronautics and Astronautics, 52958 Erciyes University, 38039 Kayseri, Türkiye; # Erciyes University Nanotechnology Application and Research Center (ERNAM), 38039 Kayseri, Türkiye; ∇ Energy Conversion Research and Application Center (ECRAC), 52958 Erciyes University, 38039 Kayseri, Türkiye; ○ BATARYASAN Enerji San. ve Tic. A. Ş., Erciyes Teknopark, Yıldırım Beyazit Mah., Aşik Veysel Bul. No: 63/B, Melikgazi, 38010 Kayseri, Türkiye

## Abstract

Polymer electrolyte membrane water electrolyzer (PEMWE) systems hold great potential for producing clean hydrogen through water electrolysis. The anode porous transport layer (PTL) is crucial to PEMWE performance; however, traditional manufacturing methods are both costly and complex. Additive manufacturing (AM), particularly selective laser melting (SLM), offers a promising alternative to enhance PTL efficiency. This study examines Cr-interlayered Pt-coated stainless-steel (SS) electrodes produced via AM, emphasizing their structural, surface, and electrochemical properties. The SS_CrPt-1.00 electrode achieved a high current density of 277.587 mA cm^–2^ at 1.9 V, with a 50-fold increase in surface area to 3.1292 cm^2^. Electrochemical impedance spectroscopy (EIS) analysis revealed a low charge transfer resistance (*R*_ct_) of 0.354 kΩ. At the same time, Tafel tests indicated a 96% reduction in the corrosion rate, resulting in a corrosion resistance of 406.011 mm year^–1^. Additionally, single-cell PEMWE experiments have demonstrated that the 3D-printed PTL structures deliver promising performance comparable to that of commercial counterparts. These in-cell evaluations confirm the practicality and applicability of the developed architecture for real operating conditions. The integration of AM-enabled PTL geometries into PEMWE systems highlights a strong potential for advancing next-generation, efficient, and economically viable hydrogen production technologies.

## Graphical abstract

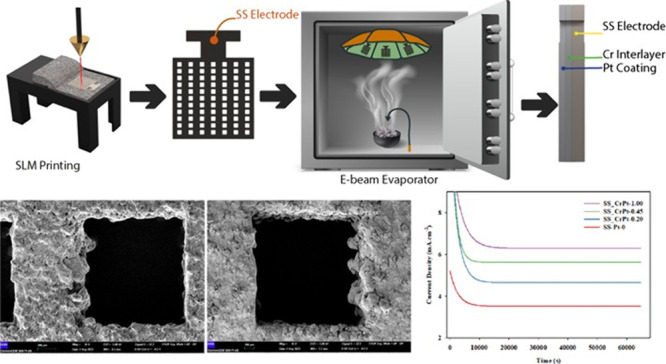

## 1 Introduction

In recent years, decision makers have increasingly focused on the renewable energy sector due to rapidly changing climate conditions. Electricity generated from intermittent energy sources such as wind and solar energy is insufficient to meet the energy demand. Hydrogen is expected to play a crucial role as an energy carrier to utilize all the energy produced.^1^ PEMWE systems are becoming more popular for hydrogen production. However, production costs of the system must be reduced to achieve commercial viability.^2–4^ The U.S. Department of Energy (DOE) has launched the “hydrogen shot” initiative to advance the development of clean hydrogen technologies, aiming to produce clean hydrogen at a cost of $1/kg in 10 years.^5^ In this technology, the anode PTL plays a critical role and is a significant contributor to the overall cost of the cell.^6^ PTL features a porous structure that facilitates interaction between the electrode and the electrolyte, enabling the transport of liquid water to the electrode surface and the rapid delivery of gas to the flow field. This structure enhances the efficiency of electrochemical reactions by promoting gas diffusion on the electrode surface.^7^ Additionally, the PTL maintains the chemical and mechanical stability of the electrode, thereby improving its long-term performance. The design and material selection of the PTL directly affect the efficiency, durability, and cost of the PEMWE. Therefore, the impact of advanced technologies (including the development of nanomaterials, polymeric membranes, and catalysts) on the PTL is continuously being investigated. Research on PTL properties and development plays a crucial role in advancing the hydrogen economy. PTLs are commercially mass-produced PEMWE equipment that may not be easily accessible. Commercial geometries may limit researchers and users from conducting diverse studies.^8^ The use of AM techniques in electrochemical systems represents a significant advancement. This progress will enable the production of lighter and more cost-effective PEMWEs through AM.^8^ Employing AM techniques such as SLM in PTL production can significantly enhance PEMWE performance. SLM offers distinct advantages over conventional manufacturing methods, particularly in producing intricate structures with precise control of fine details. SLM creates a layer-by-layer structure by melting metal powders or filaments using laser energy, enabling the fabrication of highly complex and optimized PTL structures. Key benefits of this method include high performance, precision design, diversified material options, and cost-effectiveness. Therefore, SLM-fabricated PTLs can positively enhance the efficiency, durability, and commercial viability of PEMWEs. In this context, the benefits of SLM in PTL production promote further research and application areas, significantly contributing to the development of the hydrogen economy. Numerous studies in the literature have utilized AM to produce PEMWE equipment, typically focusing on bipolar plates (BP).^9–14^ However, advancements in SLM technology enable the production of the more complex PTL structures for PEMWE.

This study examined the applicability of the electrodes fabricated for PEMWE by conducting an in-depth characterization of the PTL produced via SLM using SS316L powder, focusing on both electrochemical processes and physical properties. The designed geometry was coated with Pt at various thicknesses (0.25, 0.5, and 1 μm) using the electron beam (E-beam) evaporation method. Long-term *ex situ* tests were conducted to simulate the PEMWE anode environment and determine the corrosion resistance and durability of the PTLs. Following electrochemical and stability testing, the electrode exhibiting the best performance was evaluated in a single-cell PEMWE setup, and polarization curves were recorded. The results of this study were also compared with those of commercially available electrodes. The findings indicate that SLM exhibits promising corrosion resistance and sufficient performance, suggesting that SLM may offer a cost-effective approach for manufacturing PTLs in PEMWE systems.

## 2 Materials and Methods

### 2.1 Design and Manufacturing of PTLs

Our previous studies examined PTL geometries numerically and experimentally to identify the best PTL geometries among circle, triangle, square, and rhombus patterns. Figure 1 shows different PTL designs.^15^

**Figure 1:**
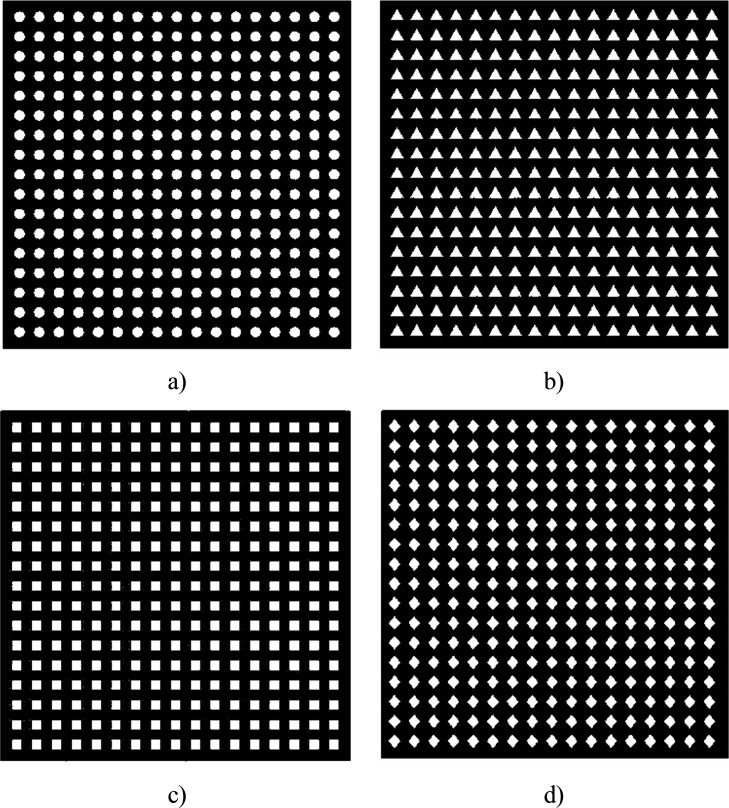
PTL geometries: (a) circle, (b) triangle, (c) square, and (d) rhombus.^15^

The flow behavior of PTLs for different patterns was investigated both numerically and experimentally at various flow rates of 100, 200, and 300 mL min^–1^. In pressure drop experiments, square PTL exhibited the most promising performance. To validate the experimental results, a numerical analysis of multiphase flow conditions was conducted. At a flow rate of 100 mL min^–1^, the square PTL geometry demonstrated a 38.96% reduction in pressure loss by directing the flow toward the outlet.^15^ Thus, square PTLs were selected as the electrode geometry for this study. PTL samples were designed using SolidWorks CAD software. The pore volume ratio of the electrodes was arranged as 25%. Two different PTL sizes were produced for use in both *in situ* and *ex situ* tests. The designed and manufactured PTL samples are shown in Figure 2.

**Figure 2:**
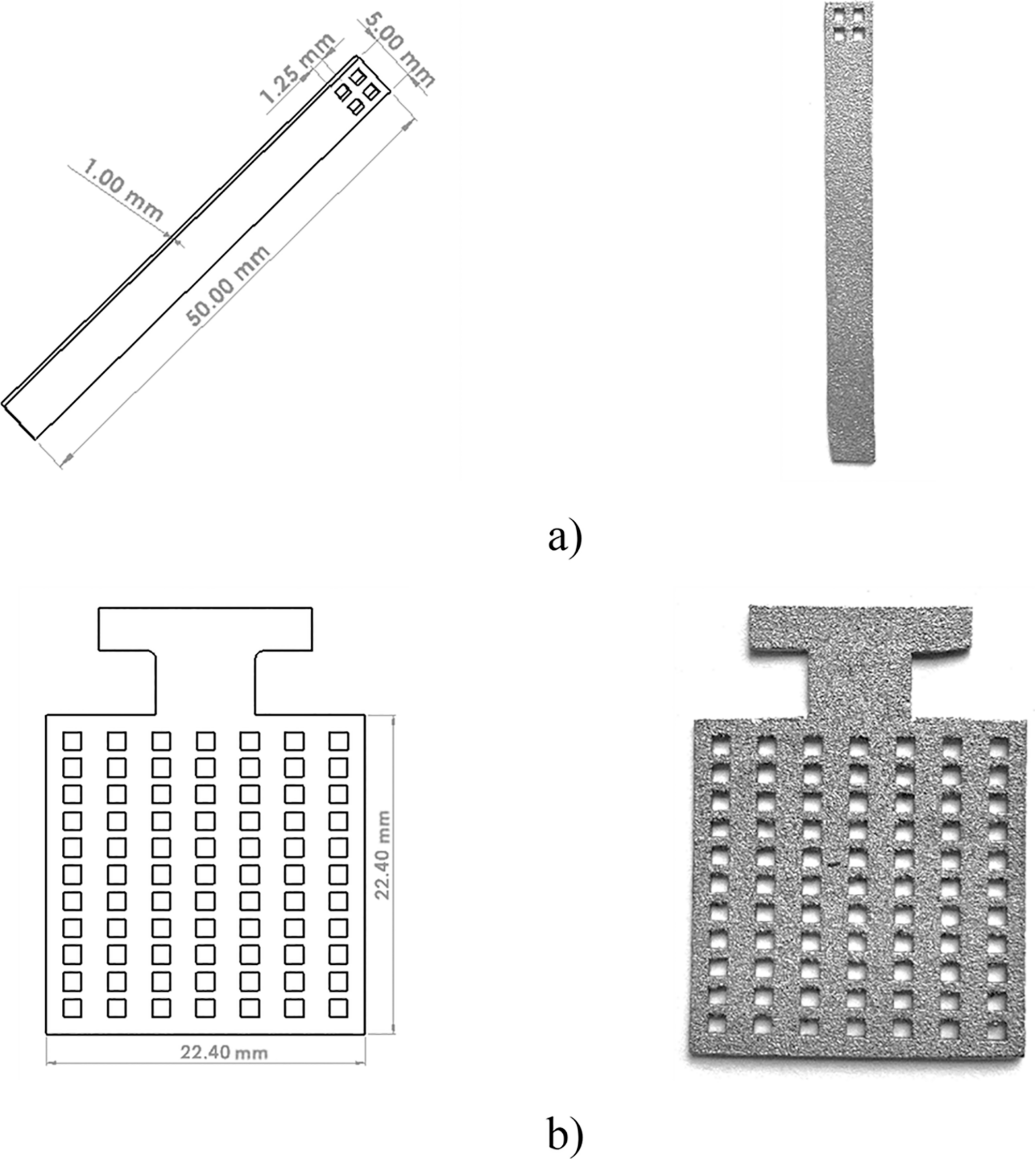
Designed and manufactured PTL samples for (a) *ex situ* electrochemical tests and (b) *in situ* corrosion and single-cell tests.

The designed geometries were manufactured using the SLM 3D printing method on an EOS M290 machine with SS316L powders. During PTL fabrication, the laser parameters (power and scan speed) were set to 195 W and 1083 mm s^–1^, respectively. The hatch distance was fixed at 0.09 mm, and the layer thickness was set to 20 μm. Before starting production, the building platform was preheated to 80 °C.

To coat the PTL samples, the E-beam evaporation method was chosen to obtain a homogeneous coating of high-melting-point Pt metal.^16^ Furthermore, the capacity to sequentially deposit multiple materials within a single vacuum cycle offers substantial benefits in the fabrication of multilayered structures, thereby enhancing adhesion and overall coating stability.^17^ Figure 3 shows a schematic representation of the coating process.

**Figure 3:**
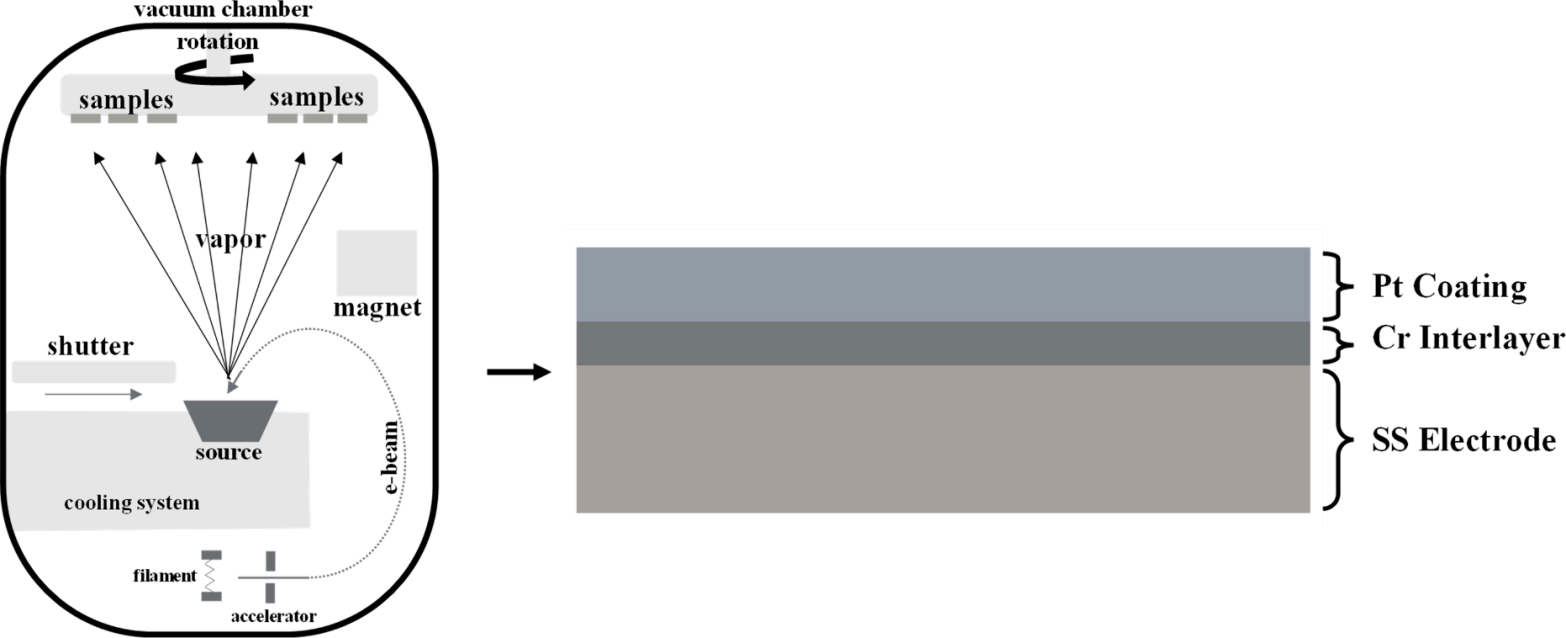
Coating process of SLM electrodes.

Pt was deposited onto the samples using the E-beam method at varying thicknesses. To improve the adhesion of the Pt film coating to the electrode surface, a thin Cr interlayer of 20 nm thickness was applied.^18,19^ To assess the adhesion effect and performance of the Cr interlayer, coatings were applied both with and without the Cr interlayer. The electrode coating conditions are listed in Table 1.

**Table 1:** Coating Conditions of SLM Electrodes

	interlayer	coating	metal loading
SS_Pt-0			
SS_Pt-0.20		0.20 μm Pt	0.43 mg_Pt_ cm^–2^
SS_CrPt-0.20	20 nm Cr	0.20 μm Pt	0.014 mg_Cr_ cm^–2^ and 0.43 mg_Pt_ cm^–2^
SS_CrPt-0.45	20 nm Cr	0.45 μm Pt	0.014 mg_Cr_ cm^–2^ and 0.97 mg_Pt_ cm^–2^
SS_CrPt-1.00	20 nm Cr	1.00 μm Pt	0.014 mg_Cr_ cm^–2^ and 2.15 mg_Pt_ cm^–2^

Pt and Cr films were deposited on SLM-printed PTLs using E-beam evaporation (NANOVAK NVEB-600) in the clean room at Erciyes University Nanotechnology Research Center (ERNAM). Prior to deposition, the SLM-printed PTL substrates were thoroughly cleaned by ultrasonic bathing for 10 min each in DI, ethanol, acetone, and isopropyl alcohol in an ultrasonic bath. They were then dried with N_2_. The clean SS substrates were placed on the substrate holder and positioned vertically with respect to the source material inside the vacuum chamber. The graphite crucible was loaded with Pt (Plasmaterials, ^1^/_8_″ diameter × ^1^/_8_″ length) and Cr (Kurt J. Lesker, 0.8 × 6 mm diameter) pellet source materials. The chamber was evacuated using a rotary pump and a turbomolecular pump to maintain a base pressure of 4 × 10^–6^ Torr. During the deposition process, the chamber pressure increased to 9 × 10^–5^ Torr, due to the release of O_2_ from the source material. In this study, the deposition rate was monitored using digital thickness monitoring systems with QCM sensors, operating at approximately 0.5 Å s^–1^. First Cr pellets and then Pt pellets were vaporized and deposited onto the electrodes by bombardment in a single vacuum run.

### 2.2 Characterization of Surface Morphologies and Crystal Structures

The surface morphology of the PTL electrodes was analyzed using a field emission scanning electron microscope (FE-SEM, Zeiss Gemini 500) equipped with an InLens electron detector. Additionally, the elemental composition of the electrodes was investigated using energy-dispersive X-ray spectroscopy (EDX) integrated with the FE-SEM system. The phase and crystal structures of the electrodes were identified through X-ray diffraction (XRD) analysis using a Bruker AXS D8 diffractometer equipped with a Cu Kα radiation anode (λ = 0.15406 nm) at a scan rate of 0.02° min^–1^.

### 2.3 Electrochemical Performance of PTL Samples

Cyclic voltammetry (CV), linear sweep voltammetry (LSV), and EIS were employed to investigate the electrochemical properties of Pt-coated PTLs. A potentiostat–galvanostat (Autolab PGSTAT204) with a three-electrode configuration was used to perform electrochemical measurements of all electrodes.^20–22^ To ensure all electrodes had the same coating area, the uncoated regions of the square PTL working electrode were insulated with Teflon tape. A 3D-printed square PTL was used as the working electrode, an Ag/AgCl (3 M saturated KCl) as the reference electrode, and a Pt wire as the counter electrode. Electrochemical measurements were conducted in a N_2_ environment using a 0.5 M H_2_SO_4_ solution. CV measurements were performed between –0.2 and 1.3 V at a scan rate of 50 mV s^–1^, and LSV measurements between –1 and 1.9 V at a scan rate of 10 mV s^–1^. EIS measurements of the PTL electrodes were conducted over a frequency range of 0.1 Hz to 10 kHz with a 5 mV amplitude and performed at open circuit potential (OCP) conditions.^23^ It should be noted that all potential reported in this study were measured without *iR* compensation.

Moreover, it is widely recognized that a high *C*_dl_ value indicates a large electrochemically active surface area. To evaluate the active surface area, the *C*_dl_ values of the electrodes were compared.^24^ It is believed that the decrease in *C*_dl_ values with increasing overpotential affects the electrochemically active surface area (ECSA) of the electrode and that hydrogen evolution reaction (HER) and oxygen evolution reaction (OER) cause clogging of the electrode pores by gas bubbles at more negative/positive overpotentials.^25^ The ECSA of the fabricated electrodes was determined by measuring the electrochemical *C*_dl_ at a non-faradaic potential. The ECSA was calculated using eq 1

1 $$\mathrm{ECSA}=\frac{C_{\mathrm{dl}}}{C_{s}}$$

where *C*_dl_ is the double-layer capacitance and *C*_s_ is the specific capacitance of the material. In this study, a standard value of 40 μF cm^–2^ was used for the specific capacitance of the metal surface.^26–28^ The measurements were conducted using a three-electrode setup in 0.5 M H_2_SO_4_ solution. CV scans were performed in a potential window of 0.4–0.7 V vs Ag/AgCl, where no faradaic reactions occur, at various scan rates (e.g., 10, 20, 30, 40, and 50 mV s^–1^).^29,30^ To determine the *C*_dl_, the difference between anodic and cathodic current densities (Δ*j* = *j*_a_ – *j*_c_) at the center of the potential window (0.55 V) was plotted against the scan rate. The slope of the resulting linear regression corresponds to twice the double-layer capacitance (slope = 2*C*_dl_). Finally, the ECSA values were obtained by dividing the calculated *C*_dl_ by the specific capacitance *C*_s_.

### 2.4 Long-Term Stability Test of PTL Samples

Tafel measurements were conducted at a scan rate of 10 mVs^–1^ between –0.3 and 0.3 V vs Ag/AgCl.^21^ Tafel polarization measurements for SS electrodes were performed using a potentiostat-galvanostat (Autolab-PGSTAT204) system in a conventional three-electrode cell configuration. The experimental data were recorded and evaluated using NOVA 2.1.5 software. The corrosion potential (*E*_corr_) and corrosion current density (*I*_corr_) were determined by fitting curves. The experimental setup used for corrosion tests is shown in Figure 4.

**Figure 4:**
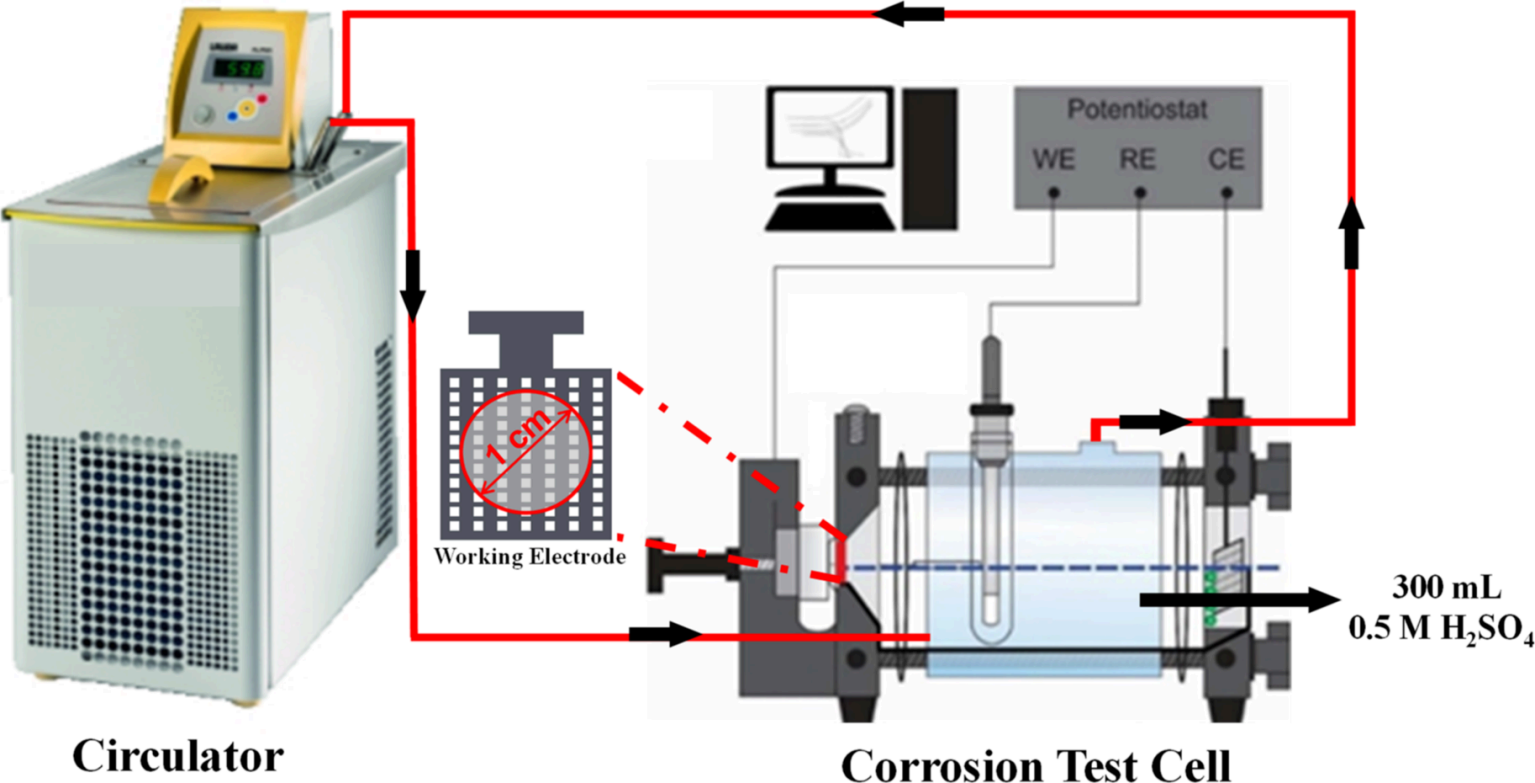
Experimental setup of corrosion tests.

In the corrosion test cell, the working electrode consisted of samples coated in a 0.5 M H_2_SO_4_ solution (fresh solution used in all tests) with a volume of 300 mL in the cell. The counter electrode was a Pt mesh (4 cm^2^), and the reference electrode was Ag/AgCl. Accordingly, 3.14 cm^2^ of coated samples (1 cm diameter region) were used as PTL samples. The solution was saturated with O_2_ by exposing it to pure O_2_ for 20 min to simulate OER environment in PEMWEs. Potentiodynamic tests were conducted at 80 °C using a potentiostat, and the results were compared between uncoated PTL structures and Pt-coated structures.^31–33^ O_2_ saturation in drop form was maintained throughout the tests. To determine the stabilization of the materials, CA tests were performed at 2 V for 18 h, and current–time (*I*–*t*) curves were recorded for each PTL sample.^34^

The physical structures and atomic ratios of the elements in the corrosion zones on the PTL sample surfaces were determined through FE-SEM and FE-SEM/EDX analysis. At the conclusion of each test, the presence of Pt structures on surfaces coated with various materials, as well as the presence of Fe, Cr, Ni, and Mo in the liquid electrolyte, was investigated. These elements constitute the primary content of SS316L material. The ICP-MS technique was used to determine the amounts of elements in the liquid at ppm levels. This study reveals the extent to which metal dissolutions and transitions can reduce the proton conductivity of the membrane and poison the Pt catalyst on the cathode side. It also investigates how different coating materials and thicknesses can mitigate these effects.^35–38^

### 2.5 Single-Cell Testing of Electrodes

A laboratory-scale PEMWE test cell with a 5 cm^2^ active area was used to evaluate the in-cell performance of the examined PTL geometries and to facilitate comparison with commercial counterparts. The MEA was prepared as described in detail in the previous studies.^39,40^ During MEA fabrication, catalyst loadings of 0.5 mg cm^–2^ Pt at the cathode and 3.0 mg cm^–2^ IrO_2_ at the anode were applied. For commercial comparison, Pt-coated Ti mesh structures with thicknesses of 10 and 2 mil were utilized. To promote uniform flow distribution inside the cell, the thinner PTL was positioned on the membrane side, while the thicker PTL was placed toward the flow-field side of the cell. The test configuration used for in-cell measurements is presented in Figure 5.

**Figure 5:**
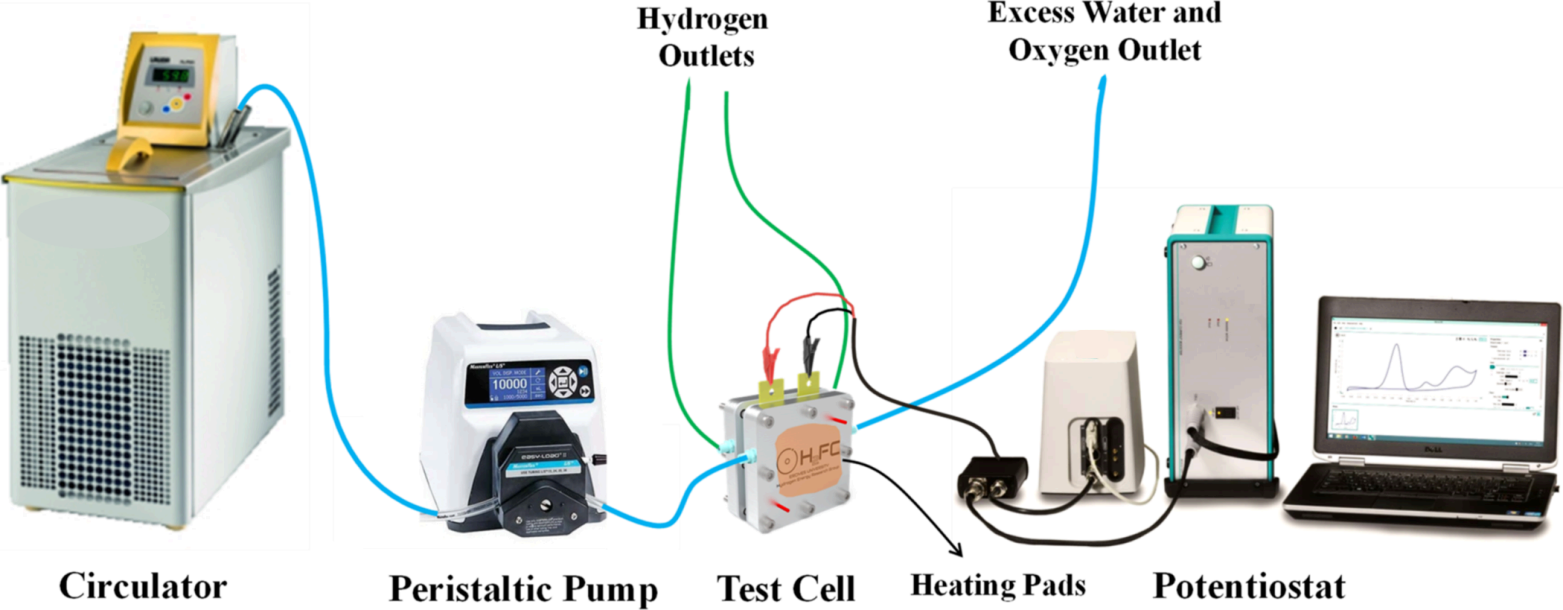
Schematic representation of the experimental setup of the PEMWE single-cell test.

Using the setup shown in Figure 5, square-shaped PTLs with an active area of 5 cm^2^ were tested. Water preheated to 80 °C was delivered to the system at a flow rate of 100 mL min^–1^ using a peristaltic pump. Heating pads placed around the cell ensured that the operating temperature was maintained at 80 °C throughout the experiments. A potentiostat (Autolab PGSTAT204) supplied the potential required to initiate electrochemical reactions. Polarization curves were recorded using the LSV method at a scan rate of 1 mV s^–1^, with a potential sweep ranging from 1.0 to 1.8 V.

## 3 Results and Discussion

### 3.1 Structural and Surface Characterizations of 3D-Printed PTLs

The XRD patterns of the SS electrodes were measured in the 2θ = 20–90° range, and the results are shown in Figure 6. In the XRD patterns of the electrode labeled SS_Pt-0, the characteristic diffraction patterns of SS316L (coded as SS) are observed.^41^

**Figure 6:**
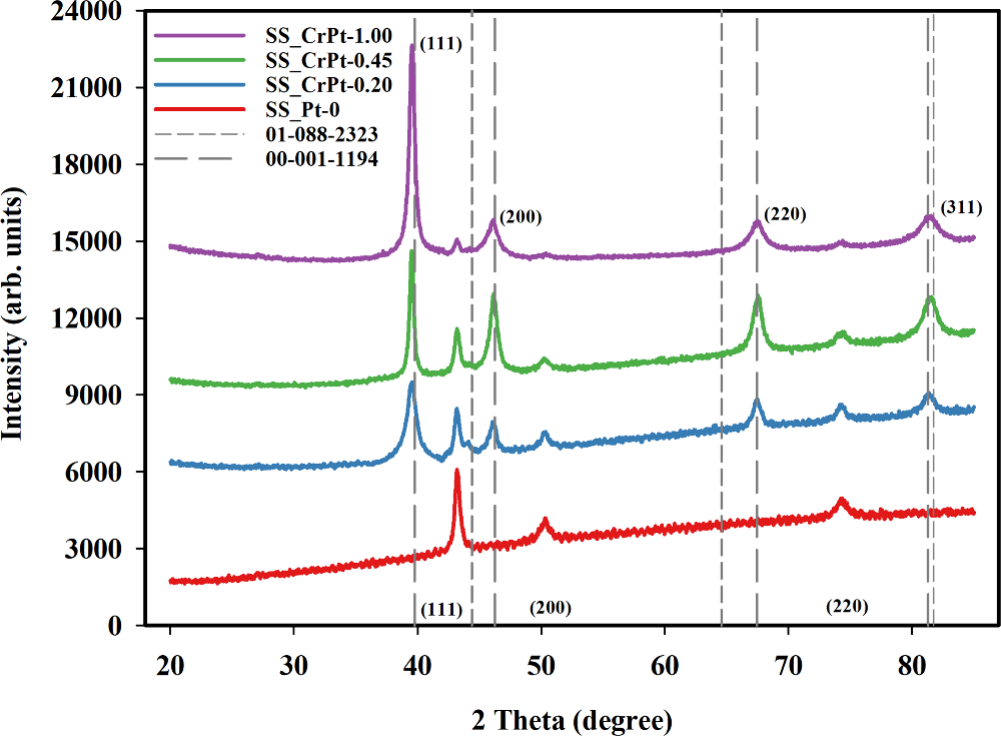
XRD diffractions of Cr-interlayered Pt-coated SS electrodes.

The XRD diffraction patterns of the uncoated and SLM-printed SS electrodes show peaks around 44°, 51°, and 75°, corresponding to the (111), (200), and (220) planes, respectively. These peaks match the diffraction patterns of SS316 material and indicate the presence of Ni, Fe, and Cr elements within the material.^42^ In the XRD patterns of the coated electrodes, peaks observed around 40°, 46°, 64°, and 82° are due to the presence of Pt. The four principal peaks, occurring at 40°, 46°, 64°, and 82°, are aligned with the crystalline structures of the (110), (200), (220), and (311) planes, respectively. Additionally, these peaks become more pronounced as the coating thickness increases. All the peaks observed in the XRD patterns of the coated SS electrodes match the CPDS card numbers 00-001-1194 and 01-088-2323.^43^ As shown in Figure 6, the diffraction patterns of the coated samples in the SS_CrPt series closely match the characteristic reflections of FCC Pt, indicating the successful formation of a polycrystalline Pt catalyst layer. A closer examination of the diffractograms reveals a subtle shift of the Pt peaks toward higher 2θ values, slightly approaching the reference positions of Cr when compared with the standard pure Pt pattern. Such minor peak shifts are commonly associated with lattice distortions and may arise from interfacial strain introduced by the underlying Cr interlayer, as well as from limited atomic interactions at the Pt–Cr interface. These observations point to a strong interfacial coupling between the adhesion layer and the Pt catalyst, which is in good agreement with the enhanced structural stability observed during the electrochemical performance tests. According to the results, no oxide form of Pt was observed in any of the coated PTL samples. It was concluded that the peak intensity increased with increasing coating thickness. Furthermore, the SS316L peaks gradually decreased with the applied coating. FE-SEM images and FE-SEM/EDX analyses of the 3D-printed PTL samples after the coating process are shown in Figure 7. Additionally, representative images illustrating the cross-sectional analysis attempts are provided in Figure S1 of the Supporting Information.

**Figure 7:**
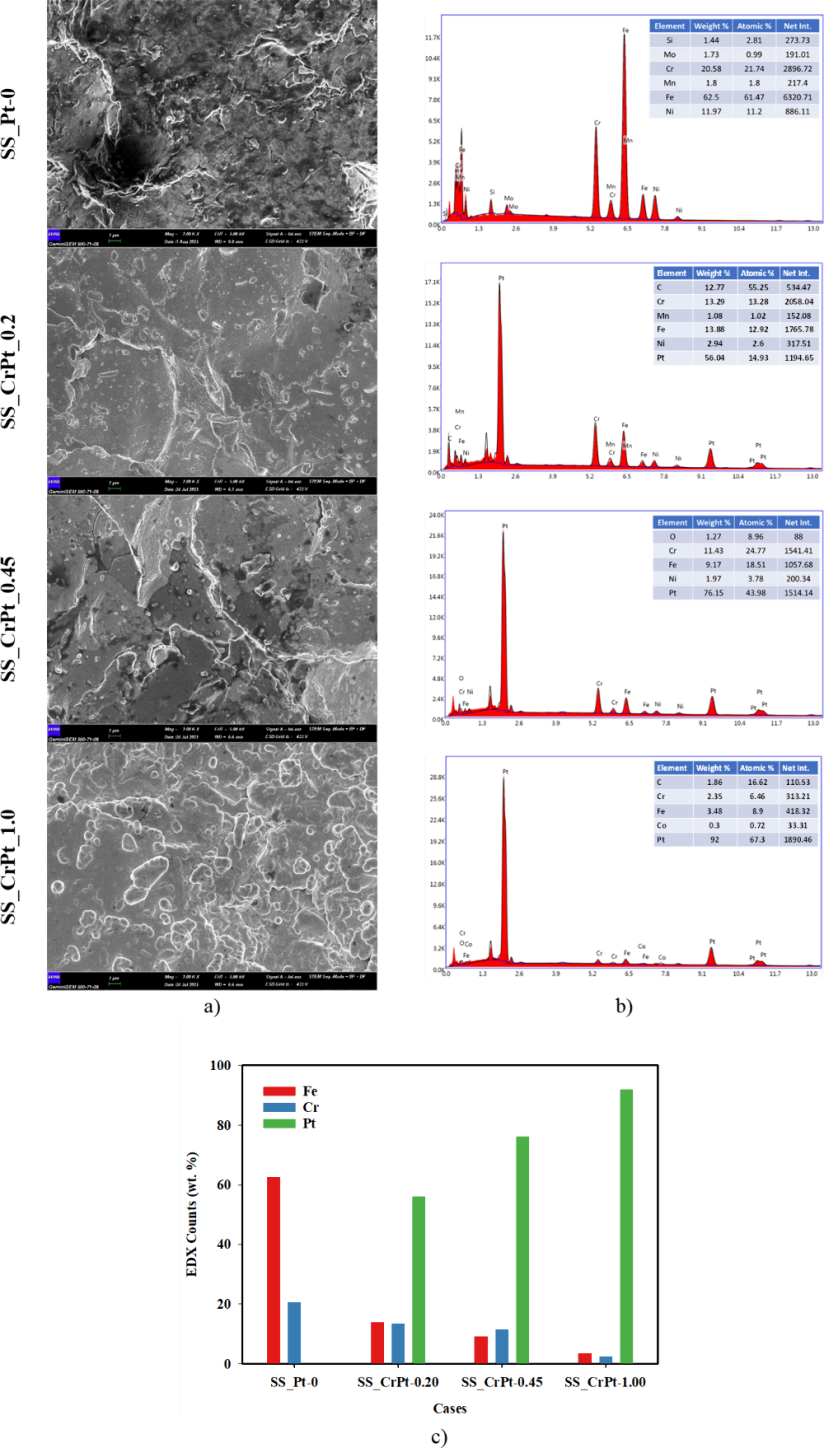
(a) FE-SEM images, (b) FE-SEM/EDX analyses of Cr-interlayered Pt-coated PTL samples, and (c) EDX counts bar graphics before the CA measurements.

When examining the FE-SEM/EDX results shown in Figure 7, the presence of Cr, Mn, Fe, and Ni elements was detected on the surface of the SS_Pt-0 electrode. These elements are inherent to the composition of SS. In the SS_CrPt-0.20 and SS_CrPt-0.45 electrodes, Cr, Fe, and Ni were observed in addition to Pt, indicating the presence of uncoated regions on the analyzed surfaces. The SS_CrPt-0.20 electrode surface exhibited 56.04 wt % Pt, while the surfaces of the SS_CrPt-0.45 and SS_CrPt-1.00 electrodes displayed Pt concentrations of 76.15 and 92 wt %, respectively. On the SS_CrPt-1.00 electrode surface, Pt, Co, Ni, and Fe were detected. The Fe concentration on the uncoated electrode surface was 62.5%, while Cr concentration was 20.58%. It was observed that the Fe-to-Cr ratio decreased as the coating was applied. The Fe ratios on the surfaces of the SS_CrPt-0.20 and SS_CrPt-0.45 electrodes were 13.88 and 9.17%, respectively, while the Cr ratios were 13.29 and 11.43%. In the SS_CrPt-1.00 electrode, the amounts of Fe and Cr were found to be 3.48 and 2.35%, respectively. However, a decrease in the number of elements other than Pt was observed. As expected, the proportion of Pt increased in accordance with the observed increase in thickness. Furthermore, the presence of elements such as Cr, Co, and Mn, which are attributable to the SS316 content, exhibited a corresponding decrease. The increase in the amount of Pt on the surface was clearly observed in the FE-SEM/EDX analyses.

Figure S1 represents FE-SEM micrographs from unsuccessful cross-sectional preparation attempts of the Pt–Cr coated SLM-fabricated SS316L PTLs. The interconnected open porosity inherent to SLM PTLs hindered reliable cross-sectioning because pores can trap mounting resin and residual air and may release volatile species under high-vacuum FE-SEM conditions.^44^ This outgassing can compromise local vacuum stability and increase beam-induced contamination, thereby reducing imaging fidelity. In addition, during embedding and sectioning, the thin-film coating locally delaminated and/or peeled off under mechanical stress (cutting, grinding, polishing), preventing reliable thickness assessment. The insulating resin also introduced pronounced charging artifacts (brightness fluctuations and image distortion).^45^

### 3.2 Electrochemical Characterizations of 3D-Printed PTLs

A Cr interlayer was applied to enhance the adhesion of the Pt coating on the electrode surface. A CA test with a duration of 18 h was conducted to investigate the effect of the Cr interlayer on Pt-coated PTL samples (Figure 8).

**Figure 8:**
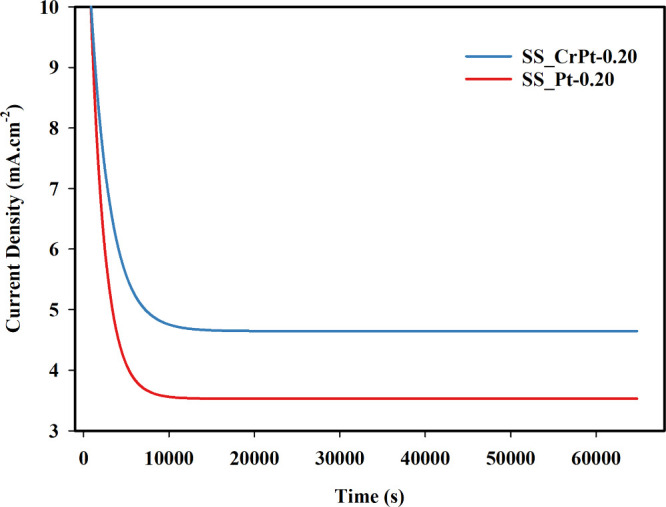
Comparison of the performance of Pt-coated PTL samples with and without a Cr interlayer.

This experiment was performed on 0.2 μm Pt coating electrodes with and without a 20 nm Cr interlayer. The CA test results showed that the application of the Cr interlayer had a beneficial effect on the electrode. In the electrode without the interlayer, the current density dropped sharply from 10 to 3.5 mA cm^–2^. However, in the electrode with the Cr interlayer, the current density decreased to 5 mA cm^–2^. This decrease in current density can be attributed to the detachment of the Pt coating from the electrode surface. The Cr interlayer enhanced the adhesion of Pt to the electrode, contributing to electrode performance by approximately 40–45%. The improved performance and durability of the SS_CrPt-0.20 sample are attributed to the critical role of the Cr interlayer acting as an adhesion promoter. Direct deposition of Pt onto stainless steel can result in weak interfacial bonding, making the catalyst prone to delamination under the mechanical stress of vigorous hydrogen gas evolution. The insertion of a Cr interlayer reduces the lattice mismatch and enhances bonding between the SS316L substrate and the Pt coating, thereby preventing physical detachment of the catalyst particles during long-term operation.^46^ Based on these results, it would be beneficial to apply Cr interlayers to Pt coatings of different thicknesses.

Figure 9 shows the results of electrochemical measurements, including CV, ECSA, LSV, and EIS.

**Figure 9:**
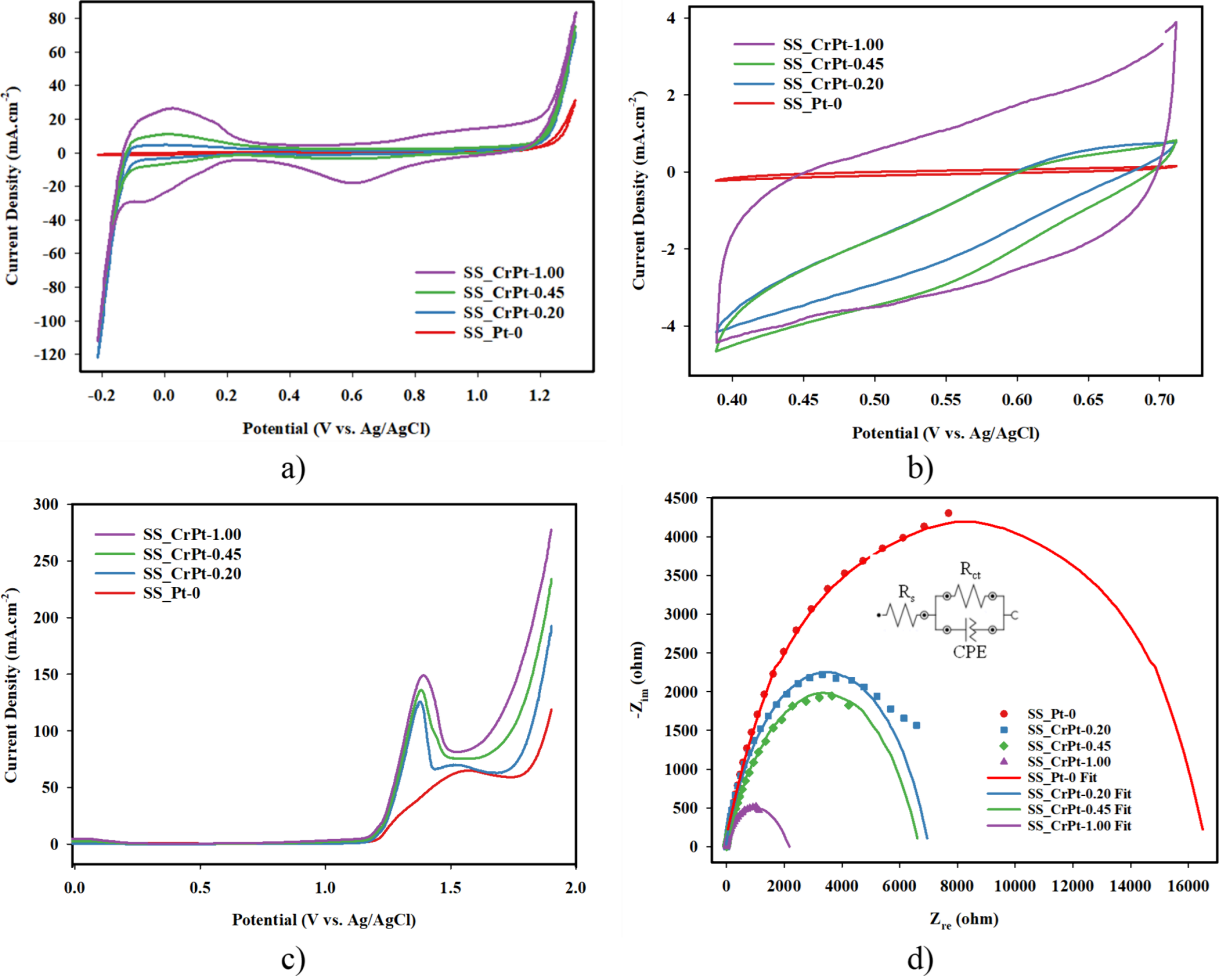
Cr-interlayered Pt-coated SS electrodes: (a) CV measurement, (b) ECSA analysis, (c) LSV measurement, and (d) EIS analysis.

Figure 9a shows the CV measurement results for Pt-coated PTL samples with a Cr interlayer. When examining these results, no peaks are observed for the uncoated SS_Pt-0 electrode, while typical Pt adsorption and desorption peaks can be observed in the coated electrodes. H_2_ desorption occurs in the positive current region around –0.10 V, while oxidation reactions occur around 0.8 V. In the reverse reaction, reduction occurs around 0.6 V, and H_2_ adsorption occurs around –0.20 V.

When examining the CV results, a current density of 76.466 mA cm^–2^ was measured at 1.3 V for the SS_CrPt-1.00 electrode. In addition, current densities of 67.378, 64.184, and 25.2903 mA cm^–2^ were obtained for the SS_CrPt-0.45, SS_CrPt-0.20, and SS_Pt-0 electrodes, respectively. As expected, the increase in Pt coating improved the electrode performance.

Figure 9b and Table 2 provide information about the ECSA of Pt-coated PTL samples. When examining the CV and ECSA results together, a parallel effect was observed between the amount of Pt coating and the ECSA. As the amount of coating increased, the ECSA also increased. As shown in Table 2, the ECSA was measured as 3.1292 cm^2^ for the SS_CrPt-1.00 electrode, while it was measured as 0.0662 cm^2^ for the uncoated electrode. When comparing these two electrodes, it can be said that the ECSA increased by approximately 50 times.

**Table 2:** ECSA Analysis of SS Electrodes

	*C*_dl_ (mF cm^–2^)	ECSA (cm^2^)
SS_Pt-0	0.0265	0.0662
SS_CrPt-0.20	0.1648	0.8241
SS_CrPt-0.45	0.2405	1.2025
SS_CrPt-1.00	1.2517	3.1292

When examining the LSV graphs in Figure 9c, the uncoated SS electrode exhibits a current density of 118.737 mA cm^–2^ at 1.9 V. At the same voltage, the SS_CrPt-1.00 electrode, which shows the best performance, reaches a current density of 277.587 mA cm^–2^. Significant increases in current were observed in the LSV results of the electrodes around 1.5 V. The peaks at this voltage are attributed to the presence of Cr. The graphs also show that the OER onset potential improves with the coating. These results indicate that the coatings enhance the kinetic activity of the 3D-printed PTL samples. Considering that PEMWEs operate between 1.6 and 2 V, it is expected that the produced electrodes will perform well within this voltage range.

Figure 9d shows the EIS analyses performed over a frequency range of 0.1 Hz to 10 kHz. As observed from the analysis results, the uncoated SS_Pt-0 electrode exhibited high resistance in the high-frequency region. In the electrodes with Pt coating, the resistance in the high-frequency region decreased. The SS_CrPt-1.00 electrode, which had the highest Pt coating thickness, exhibited the lowest resistance in the high-frequency region. These findings indicate that resistance decreases as the coating increases. Consequently, it was concluded that the Pt coating increased the conductivity and decreased the resistance on the electrode surface. The equivalent circuit parameters determined by EIS analysis are presented in Table 3.

**Table 3:** Parameters of the Equivalent Circuit from EIS Analysis

	*R*_s_ (Ω)	*R*_ct_ (kΩ)	CPE (×10^–6^ s^a^ Ω^–1^ cm^–2^)
SS_Pt-0	1.9164	11.214	0.974
SS_CrPt-0.20	1.9247	1.028	0.899
SS_CrPt-0.45	1.9428	0.786	0.868
SS_CrPt-1.00	1.9331	0.354	0.75166

The EIS analysis of SS electrodes was modeled using an equivalent circuit comprising the solution resistance (*R*_s_), charge transfer resistance (*R*_ct_), and a constant phase element (CPE) connected in parallel with *R*_ct_.^47^ All EIS tests were conducted using a freshly prepared 0.5 M H_2_SO_4_ solution. Consequently, the *R*_s_ values were very similar. The *R*_s_ value reflects the ohmic losses resulting from the solution utilized in the junction employed for the analysis and was determined to be approximately 1.9 Ω. For the SS_Pt-0 electrode, the *R*_s_ and CPE values were measured as 11.214 kΩ and 0.974 × 10^–6^ s^a^ Ω^–1^ cm^–2^, respectively. These values were observed to decrease with increasing coating amounts in the SS_CrPt-0.20, SS_CrPt-0.45, and SS_CrPt-1.00 electrodes. The SS_CrPt-1.00 electrode exhibited the lowest *R*_ct_ value of 0.354 kΩ. The *R*_s_ value was determined to be 1.028 kΩ for the SS_CrPt-0.20 electrode and 0.786 kΩ for the SS_CrPt-0.45 electrode. A decrease in *R*_ct_ results in an enhancement of OER activity, leading to an increase in current density.^20^ The EIS results demonstrate that the coating processes result in a reduction of electrode resistance.

### 3.3 Corrosion Behavior of 3D-Printed PTLs

Tafel tests were conducted to investigate the corrosion mechanism of the SS electrodes. The results of the Tafel analysis are shown in Figure 10.

**Figure 10:**
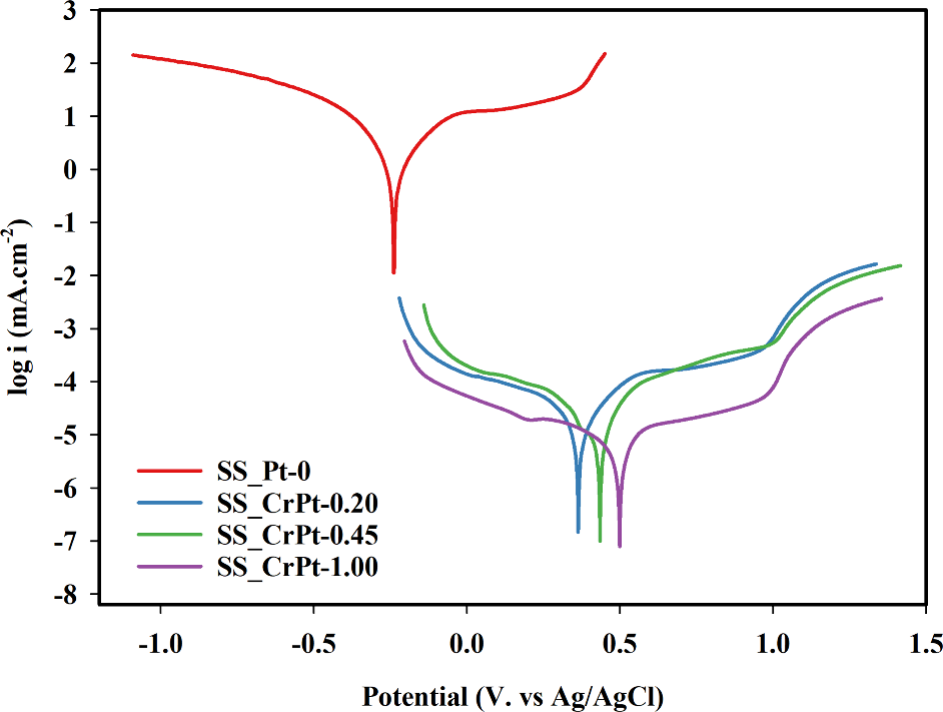
Tafel polarization curves of Pt-coated SS electrodes.

Figure 10 shows the Tafel polarization curves of the SS electrodes. The corrosion analysis results (illustrated in Figure 10) are presented in Table 4.

**Table 4:** Kinetic Parameters of 3D-Pinted SS PTLs

	*E*_corr_ (mV)	*I*_corr_ (mA cm^–2^)	β_a_ (mV dec^–1^)	*R*_p_ (Ω)	corrosion rate (mm year^–1^)
SS_Pt-0	223.84	6.251	248.51	157.587	13150.15
SS_CrPt-0.20	363.03	0.636	109.66	83.737	1337.945
SS_CrPt-0.45	433.89	0.425	87.48	60.296	894.067
SS_CrPt-1.00	498.16	0.193	46.17	31.734	406.011

Upon examining the Tafel polarization curves, it was observed that the treatments improved the corrosion resistance of the electrodes. The *E*_corr_ value of the SS_Pt-0 electrode was 223.84 mV, while those for the SS_CrPt-0.20 and SS_CrPt-0.45 electrodes were 363.03 mV and 433.89 mV, respectively. The SS_CrPt-1.00 electrode exhibited an *E*_corr_ value of 498.16 mV. It was observed that the coating processes shifted the *E*_corr_ potential, at which the electrode surface starts to corrode, toward the positive direction. This shift indicates that the electrodes can operate at higher voltages without corrosion. Furthermore, examination of the polarization resistance values showed a decrease in the *R*_p_ values of the electrodes. The *R*_p_ value for the SS_Pt-0 electrode was 157.587 Ω, while those for the SS_CrPt-0.20, SS_CrPt-0.45, and SS_CrPt-1.00 electrodes were 83.737, 60.296, and 31.734 Ω, respectively. The *R*_p_ value decreased with increasing coating thickness. This reduction is a result of coating the surface with a protective layer, which improves the corrosion rate. The corrosion rate at the SS_Pt-0 electrode with the highest *R*_p_ value was 13 150.15 mm year^–1^. In contrast, this rate was 406.011 mm year^–1^ for the SS_CrPt-1.00 electrode, indicating an improvement of approximately 96% in surface corrosion. This 96% reduction in surface corrosion effectively prevented performance degradation that may have occurred in the PEMWE system.

The results of the CA tests conducted for 18 h in a 0.5 M H_2_SO_4_ solution saturated with O_2_ at 80 °C to simulate the environment of a PEMWE using SS electrodes are presented in Figure 11.

**Figure 11:**
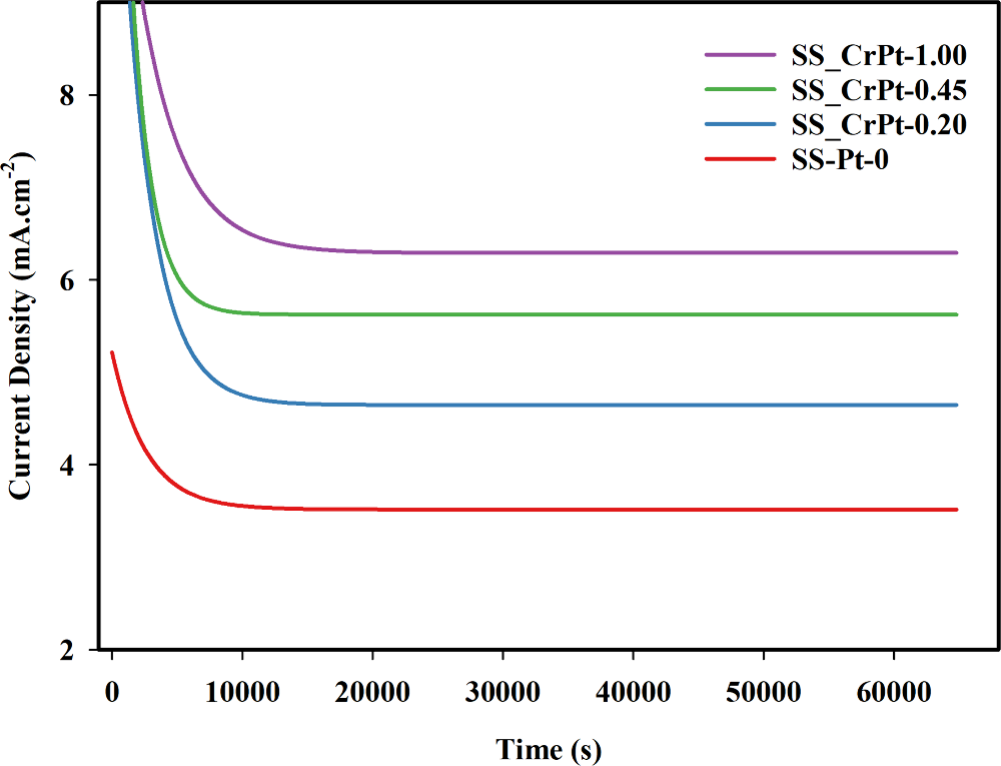
CA stability test results for Cr-interlayered Pt-coated SS electrodes.

As shown in Figure 11, the current density reached a steady state after approximately 10 000 s. Additionally, the current drawn by the coated electrodes decreased over time, likely due to the coating material dissolving into the solution under the highly corrosive conditions. The pronounced current density decay observed during this initial period can be attributed to an electrochemical conditioning process, during which the electrode surface and interfaces gradually reach a more stable state. Given the complex, rough morphology of the SLM-fabricated substrate, microscopic coating discontinuities or pores may exist locally within the deposited layers. During the early stage of operation, electrolyte access through such defects likely exposes small regions of the underlying SS316L, promoting the rapid formation of a passive oxide film and a corresponding increase in interfacial resistance. Furthermore, the intense gas evolution at the beginning of the CA test leads to surface rewetting and bubble coverage effects, alongside the possible detachment of a minor fraction of mechanically weakly adhered catalyst particles, all of which contribute to this initial transient behavior. However, even after these stability tests conducted in the highly corrosive environment, the current density values measured on the coated electrodes are much higher than those of the uncoated SS_Pt-0 electrode. After 18 h of testing, the current density of the SS_Pt-0 electrode was 3.517 mA cm^–2^. Furthermore, the current densities of the SS_CrPt-0.20, SS_CrPt-0.45, and SS_CrPt-1.00 electrodes were 4.646, 5.625, and 6.292 mA cm^–2^, respectively. Examining the CA test results of the 3D-printed PTL structures, the current density obtained after the 18-h test of the SS_Pt-0 electrode was 3.828 mA/cm^2^. Additionally, the CA test results indicate that the effects of the coating processes are maintained even after 18 h of testing. FE-SEM images taken before and after the CA test to observe the impact of stability on the surface are provided in Figure 12.

**Figure 12:**
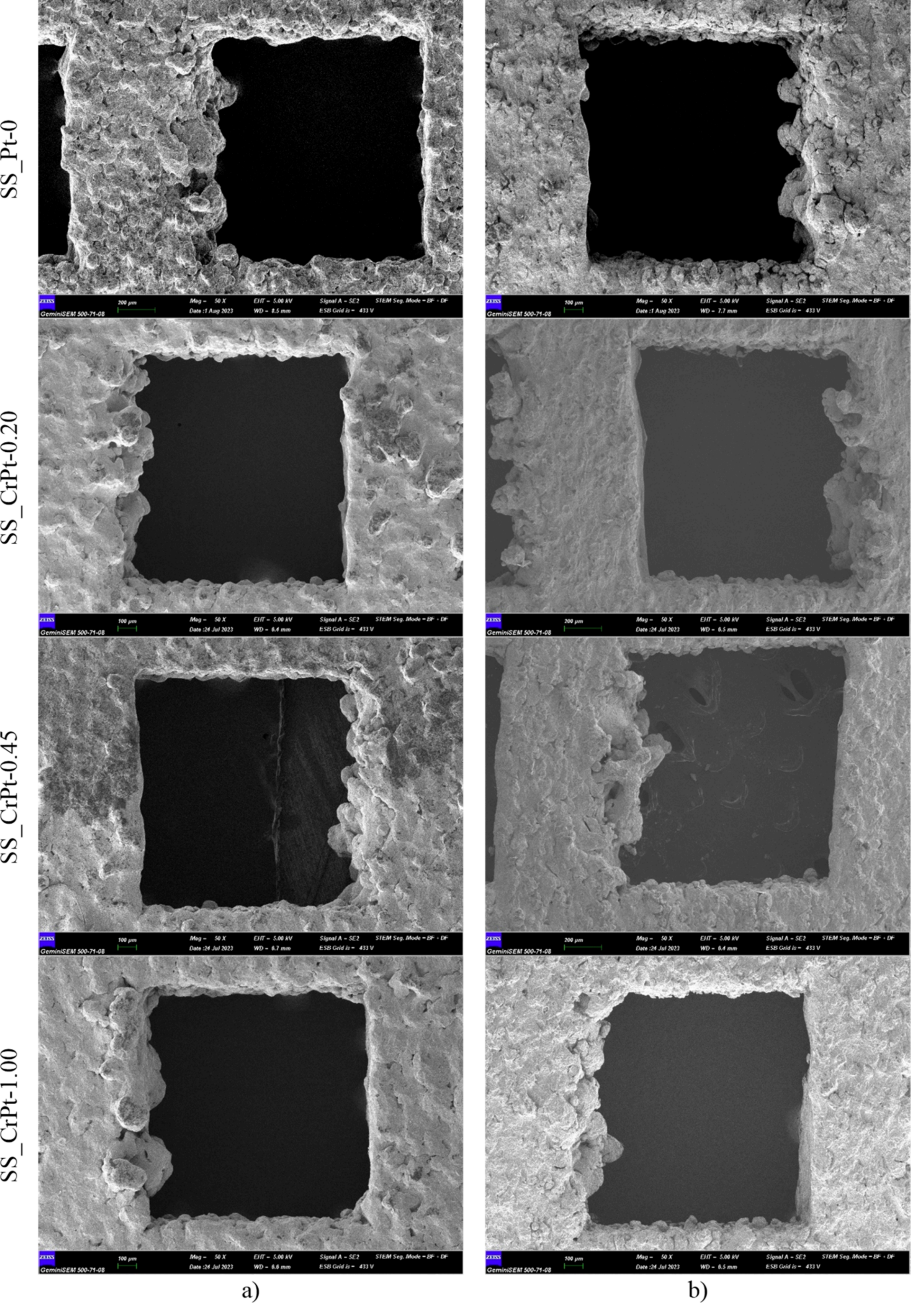
FE-SEM images of Cr-interlayered Pt-coated SS electrodes (a) before and (b) after corrosion testing.

FE-SEM images of the SS electrodes taken before (left) and after (right) the CA tests are shown in Figure 12. Partial distortions were observed on the surfaces of the SS electrodes after the stability test. However, no corrosion was detected in the relevant regions where FE-SEM images were taken. It was noted that the surface distortions on the coated electrodes before the CA tests were less severe than those on the uncoated electrodes. These findings suggest that the coatings offer effective protection for the SS surfaces. Figure 13 presents the FE-SEM/EDX analyses conducted following the stability tests simulating the PEMWE environment.

**Figure 13:**
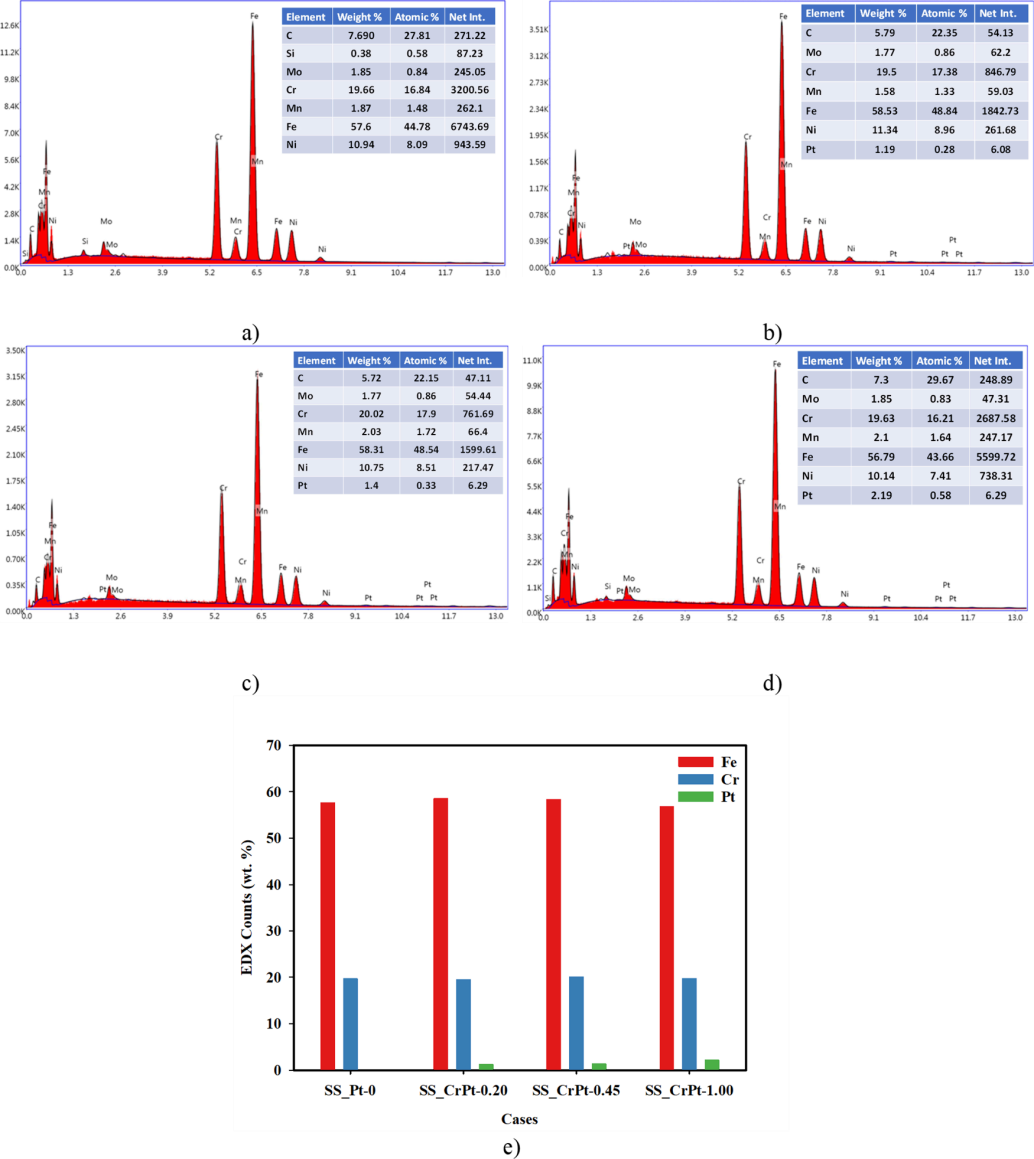
FE-SEM/EDX analyses of (a) SS_Pt-0, (b) SS_CrPt-0.20, (c) SS_CrPt-0.45, and (d) SS_CrPt-1.00 electrodes after CA testing and (e) bar graph of EDX counts after CA testing.

As shown in the FE-SEM/EDX analyses in Figure 13, even after the stability tests conducted in the highly corrosive environment, Pt remained on the surface of the coated PTL samples. According to the FE-SEM/EDX analyses conducted after the CA tests, the SS_CrPt-0.20 electrode surface contains 1.19 wt % Pt. In comparison, the Pt content on the SS_CrPt-0.45 electrode surface was measured at 1.4 wt %. On the SS_CrPt-1.00 electrode, the Pt content was found to be 2.19 wt %. These findings are consistent with the stability test results of the coated SS electrodes. It was concluded that the cause of the current drop in the CA tests was the dissolution of the Pt coating on the SS electrode surfaces over time. Additionally, the density values measured in the FE-SEM/EDX analyses confirm the density values obtained from the XRD analyses. The XRD diffraction patterns collected after the stability tests are presented in Figure 14.

**Figure 14:**
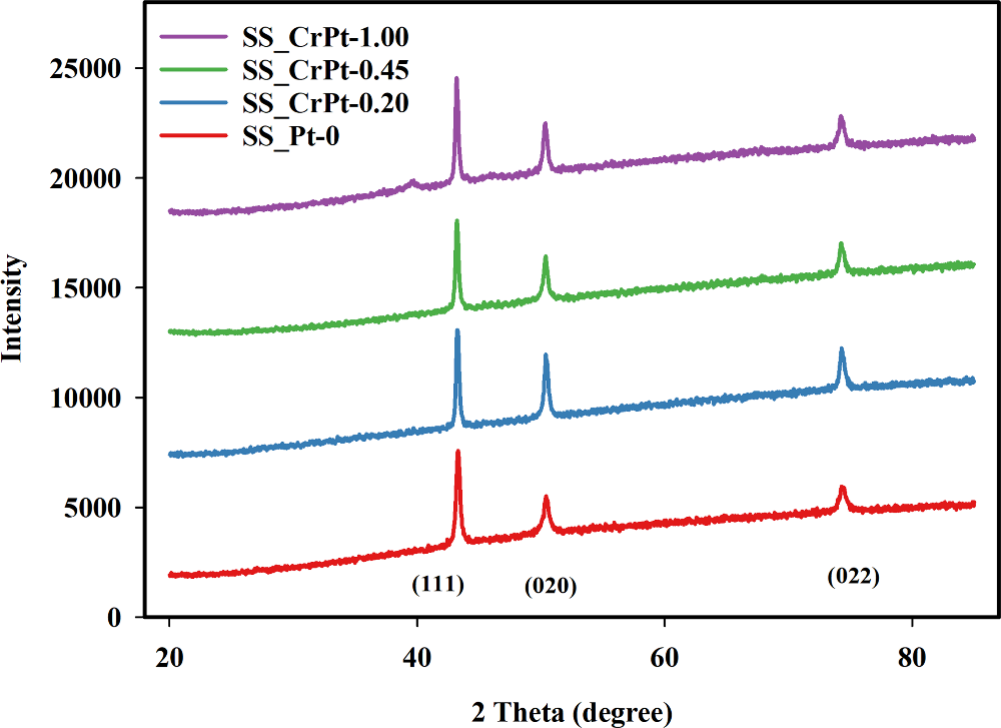
XRD diffraction patterns after CA testing.

The XRD analysis results obtained after the 18 h CA tests of the SS electrodes are shown in Figure 14. According to the analysis, the peaks observed at approximately 43°, 51°, and 76° in the XRD patterns are attributed to elements inherent in the SS material and the compounds they form with one another. These peaks correspond to the (111), (020), and (022) planes, respectively. No oxidized compounds were detected in the SS electrodes after the stability tests. A peak related to the Pt element was not observed in the diffraction patterns of the SS_CrPt-0.20, SS_CrPt-0.45, and SS_CrPt-1.00 electrodes due to the reduction in the weight percentage of dissolved components. These findings indicate that the coating thicknesses were insufficient and that a heat treatment step is required after the coatings to enable Pt to better integrate within the grain boundaries of the SS even after the stability tests. The ICP–MS analysis results obtained from the solution samples used in the stability tests are presented in Table 5.

**Table 5:** ICP-MS Analysis Results of SS Electrodes

	Cr (ppm)	Mn (ppm)	Ni (ppm)	Mo (ppm)	Pt (ppm)
SS_Pt-0	7.55	0.67	4.44	1.21	
SS_CrPt-0.20	11.98	0.98	6.78	6.78	0.01
SS_CrPt-0.45	12.38	1.01	7.03	7.03	0.02
SS_CrPt-1.00	11.97	0.97	6.67	6.67	0.03

The ICP-MS analysis results for determining the amounts of elements dissolved into the solution after the 18 h CA tests are shown in Table 5. The elements Cr, Mn, Ni, and Mo were proportionally the highest in SS coded SS316L. Cr and Pt were materials used in the coatings. It was observed that the ratio of Pt in the solution increased proportionally with the increase in Pt loading. When the amounts of elements originating from SS (Cr, Mn, Ni, and Mo) in the solution were examined, it was observed that the ratio of SS316L elements dissolved into the solution decreased as the coating thickness increased. This result provides evidence that the coating process protects the electrode surface. Furthermore, the coating supplied was sufficient for long-term tests conducted at 80 °C with O_2_ saturation for 18 h.

### 3.4 Single-Cell Testing of Electrodes

In Figure 15, the polarization curves obtained from the cell tests conducted in the voltage range of 1.0–1.8 V are presented.

**Figure 15:**
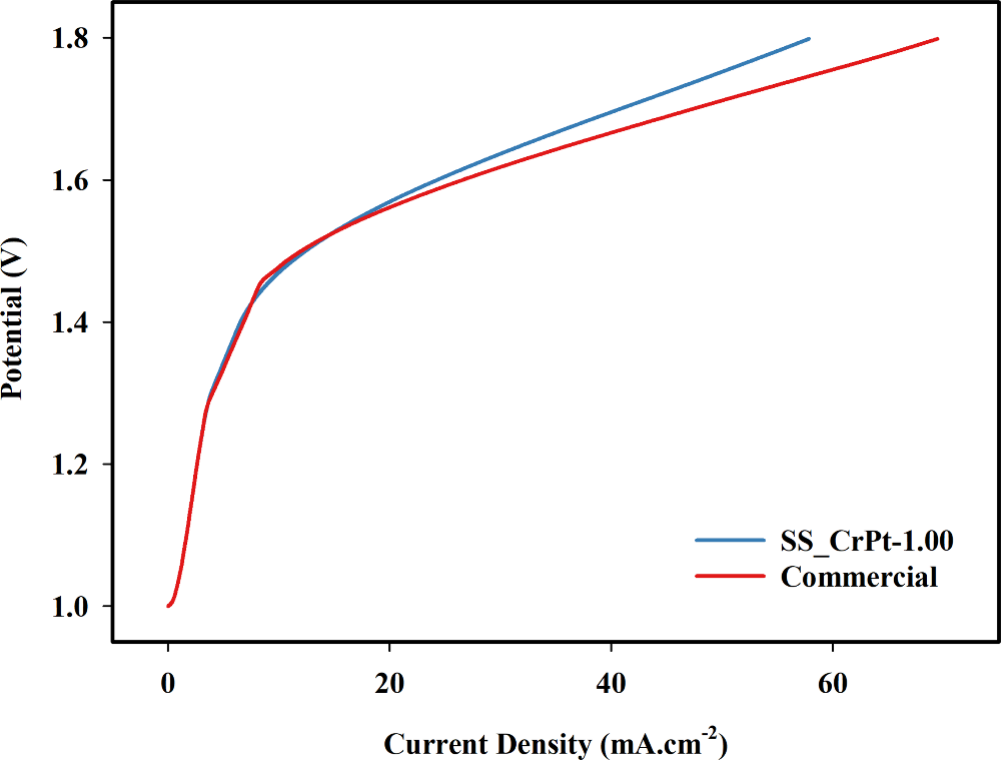
Polarization curves of single-cell tests.

The polarization curves obtained for the SS_CrPt-1.00 sample and the commercial PTL are presented in Figure 15. As shown in the figure, both structures exhibit similar activation behavior at low current densities; however, noticeable deviations emerge as the cell potential increases. These differences become increasingly pronounced in the high-voltage region, where the influence of ohmic and mass-transport losses is more dominant. At 1.8 V, the SS_CrPt-1.00 PTL delivers a current density of 57.85 mA cm^–2^, whereas the commercial PTL reaches 69.44 mA cm^–2^, indicating that the 3D-printed structure performs approximately 20% lower than the commercial counterpart under these conditions. In addition, the commercial PTL demonstrates a slightly earlier current onset in the activation region, suggesting a marginally reduced barrier for charge-transfer initiation. These differences can be attributed to the intrinsic electrical and structural properties of the materials. While the commercial PTLs are manufactured from Ti, which possesses significantly higher electrical conductivity, the SS_CrPt-1.00 sample is based on SS. Although Pt coatings are present on both PTLs, the inherent conductivity disparity between Ti and SS leads to a higher ohmic resistance in the SS-based structure, particularly at elevated current densities. Furthermore, the porosity of the SS_CrPt-1.00 PTL is approximately 25%, whereas the commercial Ti-mesh PTLs exhibit porosity values in the range of 55–65%.^48^ The higher porosity of the commercial materials facilitates more effective water transport toward the membrane-electrode interface, which becomes increasingly critical at higher operational voltages. In contrast, the lower porosity of the SS_CrPt-1.00 geometry may restrict water accessibility in the deeper regions of the structure, thereby intensifying mass-transport limitations. This effect is reflected in the divergence of the curves at higher potentials, where the slope of the SS_CrPt-1.00 profile increases more prominently. Such limitations also lead to an increase in mass transport resistance, which ultimately reduces overall cell performance at elevated voltages. Beyond performance-related factors, material cost also plays a crucial role in technology scalability. Despite employing comparable Pt-coating procedures, substituting Ti with SS316L offers a substantial reduction in material and processing costs. This economic advantage is significant for PEMWE systems, where the high price of Ti-based components remains a major barrier to widespread adoption. Achieving performance reasonably close to commercial Ti PTLs at a significantly lower cost demonstrates that 3D-printed SS316L architectures have strong potential to enhance the affordability and large-scale deployment of PEMWE technologies.

Overall, these results demonstrate that the SS_CrPt-1.00 PTL exhibits competitive performance in the intermediate voltage region (1.45–1.65 V), yet the combined effects of higher ohmic resistance and reduced porosity lead to observable performance losses at higher current densities. These findings highlight the importance of further optimization in both material selection and structural design for 3D printed PTLs intended for PEMWE applications.

## Conclusion

The results of this study demonstrate that PTLs fabricated from SS316L using SLM offer significant potential as cost-effective, efficient components for PEMWE systems. The application of a Cr interlayer followed by a Pt coating was found to be critical in enhancing both the electrochemical performance and corrosion resistance of the SS substrates. Structurally, the coating process increased the ECSA by a substantial amount. The SS_CrPt-1.00 electrode exhibited the highest ECSA of 3.13 cm^2^, representing a nearly 50-fold improvement over the uncoated SS_Pt-0 reference (0.07 cm^2^). This confirms a direct correlation between the coating amount and the availability of active sites. In terms of stability, the Cr interlayer played a pivotal role; Tafel analysis revealed that the corrosion rate decreased by approximately 96% for the SS_CrPt-1.00 sample (406 mm year^–1^) compared to the uncoated electrode (13.150 mm year^–1^). Long-term chronoamperometry tests (18 h) further corroborated this, showing that the coated electrodes maintained stable, high current densities by preserving surface-active sites in harsh oxidative environments.

Electrochemically, the functionalized PTLs demonstrated superior activity. The LSV results showed that the SS_CrPt-1.00 electrode achieved a current density of 277.6 mA cm^–2^ at 1.9 V, corresponding to a roughly 2-fold increase in performance over the uncoated sample. In single-cell PEMWE experiments, the 3D-printed SS_CrPt-1.00 PTL delivered performance comparable to that of commercial Ti-mesh PTLs, particularly in the midvoltage region. Although performance deviations were observed at higher voltages, attributed to elevated ohmic and mass-transport resistances stemming from the lower conductivity and porosity of the printed structure, the sample delivered only ∼20% lower current density than the commercial benchmark at 1.8 V.

In summary, this work highlights that SS316L-based PTLs, when modified with an appropriate Cr/Pt coating, can approach the performance levels of conventional titanium-based components while offering an inherent cost advantage. The use of stainless steel supports more economically viable component fabrication, strengthening the potential for large-scale adoption. Future efforts focusing on optimized porosity distribution, refined internal geometries, and scalability of the production process may further minimize mass-transport limitations and enhance the applicability of these additively manufactured PTLs in high-performance PEMWE systems.

## Supplementary Material



## Notes

Cross-sectional SEM limitations of porous SLM SS316L PTLs (Figure S1) (PDF)
