# Supplementary material for: Pt–Cr Coated 3D-Printed Porous Transport Layers for Proton-Exchange Membrane Water Electrolyzers Prepared by Electron Beam Evaporation
Source: Langmuir. 2026 Mar 18;42(16):10946–59. doi: 10.1021/acs.langmuir.5c06168 (PMC13130959; doi:10.1021/acs.langmuir.5c06168)
Supplement: Supplementary file 1 [file la5c06168_si_001.pdf]

Supporting Information

Pt-Cr Coated 3D-Printed Porous Transport Layers for Proton Exchange Membrane Water Electrolyzers Prepared by Electron Beam Evaporation

Murat Kıstı [1,2,3,4], Emre Özdoğan [1,2,3,4,8], Sami Pekdemir [5,6], Mehmet Fatih Kaya [1,3,4,7,8] *

[1]Erciyes University, Engineering Faculty, Energy Systems Engineering Department, Heat Engineering Division, 38039, Kayseri, Türkiye

[2]Erciyes University, Graduate School of Natural and Applied Sciences, Energy Systems Engineering Department, 38039, Kayseri, Türkiye

[3]Erciyes University H2FC Hydrogen Energy Research Group, 38039, Kayseri, Türkiye

[4]Erciyes University, ArGePark Research Building, Kayseri, Türkiye

[5]Department of Aeronautical Engineering, Faculty of Aeronautics and Astronautics, Erciyes University, Kayseri 38039, Türkiye

[6]ERNAM - Erciyes University Nanotechnology Application and Research Center, Kayseri 38039, Türkiye

[7]Energy Conversion Research and Application Center (ECRAC), Erciyes University, 38039, Kayseri, Türkiye

[8]BATARYASAN Enerji San. ve Tic. A. Ş., Erciyes Teknopark, Yıldırım Beyazit Mah. Aşik Veysel Bul. NO: 63/B Melikgazi, 38010, Kayseri, Türkiye

* Corresponding author. Tel.: +90 3522076666 / 32331.

E-mail address: kayamehmetfatih@erciyes.edu.tr

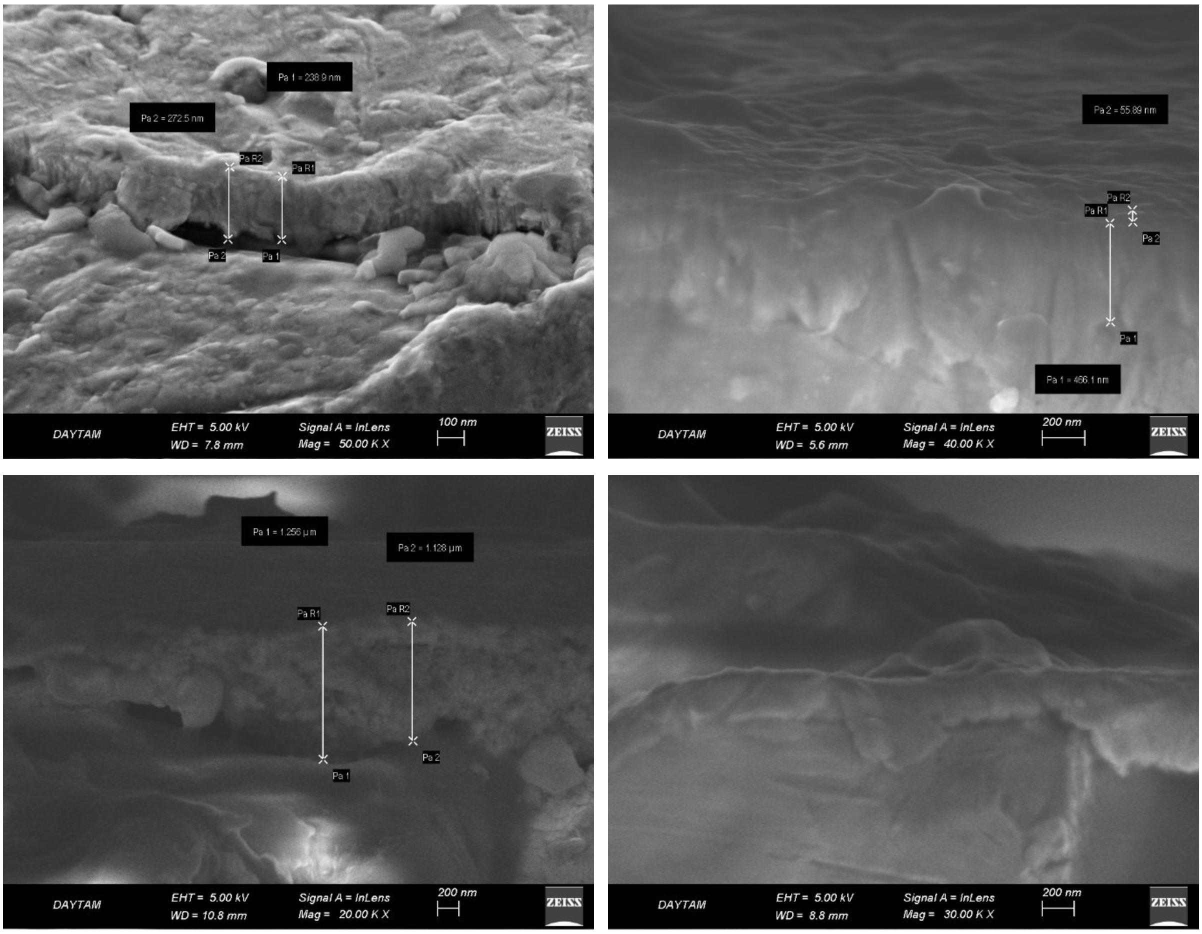

Figure S1. Cross-sectional SEM limitations of porous SLM SS316L PTLs
